# The evolution of disgust for pathogen detection and avoidance

**DOI:** 10.1038/s41598-021-91712-3

**Published:** 2021-06-29

**Authors:** Jessica K. Hlay, Graham Albert, Carlota Batres, George Richardson, Caitlyn Placek, Steven Arnocky, Debra Lieberman, Carolyn R. Hodges-Simeon

**Affiliations:** 1 Department of Anthropology, Boston University, 232 Bay State Rd. #105, Boston, MA 02215 USA; 2 Department of Psychology, Franklin & Marshall College, Lancaster, PA USA; 3 School of Human Services, University of Cincinnati, Cincinnati, OH USA; 4 Department of Anthropology, Ball State University, Muncie, IN USA; 5 Department of Psychology, Nipissing University, North Bay, ON Canada; 6 Department of Psychology, University of Miami, Coral Gables, FL USA

**Keywords:** Evolution, Psychology, Diseases

## Abstract

The behavioral immune system posits that disgust functions to protect animals from pathogen exposure. Therefore, cues of pathogen risk should be a primary driver influencing variation in disgust. Yet, to our knowledge, neither the relationship between current pathogen risk and disgust, nor the correlation between objective and perceived pathogen risk have been addressed using ecologically valid measures in a global sample. The current article reports two studies addressing these gaps. In Study 1, we include a global sample (*n* = 361) and tested the influence of both perceived pathogen exposure and an objective measure of pathogen risk—local communicable infectious disease mortality rates—on individual differences in pathogen and sexual disgust sensitivities. In Study 2, we first replicate Study 1’s analyses in another large sample (*n* = 821), targeting four countries (US, Italy, Brazil, and India); we then replaced objective and perceived pathogen risk with variables specific to the SARS-CoV-2 pandemic. In Study 1, both local infection mortality rates and perceived infection exposure predicted unique variance in pathogen and sexual disgust. In Study 2, we found that perceived infection exposure positively predicted sexual disgust, as predicted. When substituting perceived and objective SARS-CoV-2 risk in our models, perceived risk of contracting SARS-CoV-2 positively predicted pathogen and sexual disgust, and state case rates negatively predicted pathogen disgust. Further, in both studies, objective measures of risk (i.e., local infection mortality and SARS-CoV-2 rates) positively correlated with subjective measures of risk (i.e., perceived infection exposure and perceived SARS-CoV-2 risk). Ultimately, these results provide two pieces of foundational evidence for the behavioral immune system: 1) perceptions of pathogen risk accurately assay local, objective mortality risk across countries, and 2) both perceived and objective pathogen risk explain variance in disgust levels.

## Introduction

Disgust, also referred to as the “behavioral immune system” (BIS^1^; see also ^2–6^), is a suite of psychological and behavioral adaptations that evolved to mitigate the costs associated with micro-organismic infection. Disgust relies on the detection of cues of infectious agents in the immediate environment^4^, which then activates, in a context- and person-specific manner, behavioral and cognitive responses that prompt the exposed individual to engage in infection-reducing, health-protective behaviors^1^. The ability to detect pathogens prior to ingestion helps to prevent the substantial energetic burden associated with mobilizing the physiological immune system^7,8^. Because of the potential opportunity costs of infection-risk reduction (e.g., social avoidance), the BIS should be flexible, such that individuals experience more strongly aversive reactions in situations that have reliably co-occurred with increased infection risk during human evolution^4^. Thus, BIS theory predicts principled, facultative variation in response to infectious disease risk^1,4^.

The BIS and disgust likely function to help protect against several routes of disease transmission, including foods, objects, surfaces, and other people^9,10^. In addition, the mouth and genitals likewise serve as portals of contamination. For this reason, researchers have proposed that disgust functions to regulate consumption and contact behaviors of individuals, thereby mitigating risks of pathogen infection (e.g. ^11,12^). Disgust may also operate in the moral domain (e.g., ^13,14^); however, there is some debate (e.g., ^5^).

To measure sensitivities to pathogen disgust (which captures both consumption and contact behaviors), sexual disgust, and moral disgust, Tybur and colleagues^15^ developed the Three Domains of Disgust scale (TDDS). These three domains relate to pathogen avoidance in distinct ways^6, 11,16,17^. First, *pathogen* *disgust* promotes aversive reactions to potentially infectious agents and scenarios, perhaps most clearly related to the BIS^5^. For example, individuals with higher pathogen disgust are more likely to avoid people in general^18^. Second, *sexual* *disgust* is thought to adjust mating strategies by promoting aversion to sexual partners who could decrease reproductive success (e.g., siblings^19^). Those with higher sexual disgust show reduced interest in uncommitted sex^11,12^. In one study, individuals exposed to an experimental olfactory disgust condition reported higher intent to use condoms than those who had not been primed^20^. The third domain—*moral* *disgust*—seems to be elicited by individuals who violate perceived social norms. Individuals with higher disgust are more likely to perceive social distance from those with foreign accents^14^ and experience more out-group negativity^13^. The researchers suggest that these behaviors limit pathogen exposure from out-group members, who are likely to carry novel infectious threats^13,14^.

### The current research

Here we identify and address a critical gap in the literature: A central prediction of the BIS theory is that disgust should be responsive to the level of communicable infection risk in the environment^1^; however, few studies have addressed this core hypothesis. Several studies have examined infection risk and disgust in laboratory environments^18,20,21^. For example, Mortensen and colleagues^21^ found that experimentally inducing a high-pathogen condition resulted in more behavioral avoidance of disease threats. Yet only two studies have directly examined the relationship between actual, ecologically-relevant infectious disease risk and disgust levels^22,23^. Both used the Index of Disease Prevalence (IDP^24^), a historical measure that defines “parasite stress” using mid-twentieth century epidemiological maps of nine infectious diseases. Skolnick and Dzokoto^22^ found that in Ghana, which has a higher IDP, participants had higher pathogen and moral disgust levels than in the US, which has a lower IDP; however, sexual disgust was not examined in this study. This evidence supports elements of the BIS, demonstrating that those in an environment with a higher pathogen load perceive themselves to be more vulnerable to disease and react with more psychological aversion to pathogen cues. Causal inference is not warranted in the context of a comparison of only two countries, however, because it is not possible to control any potential confounders that vary between countries along with parasite stress and disgust.

Tybur and colleagues^23^ extended this analysis to include 30 countries. They found that historical pathogen load predicted traditionalism; however, it was not significantly related to pathogen disgust and associations with sexual and moral disgust were not tested. Although the IDP is a useful measure of historical pathogen load, we contend that current infection risk should be more closely aligned with a facultative infection-reducing system. Therefore, in the present study, we use recent state-level communicable disease mortality rates as indicators of objective infection risk. By examining state-level variation within countries, we are also able to account for between-country differences (e.g., cultural practices) that might confound our findings—a limitation of Skolnick and Dzokoto’s^22^ study. Additionally, if disgust evolved to protect against pathogen transmission, then we should observe this relationship across relevant domains; therefore, we test the associations between current infection risk and pathogen and sexual disgust to extend the findings of Skolnick and Dzokoto^22^ and Tybur et al.^23^.

Further, because the BIS is hypothesized to have evolved to respond to perceived pathogen cues, perception should be a trusted mechanism of detection of actual pathogen risk. Previous research shows that individuals reliably perceive cues of infection in others through olfaction^25,26^, appearance^26,27^, and body motion^28^. However, previous work has been experimental, and the association between perception and objective infection cues in humans has yet to be studied in a natural environment. To explore this prediction in the current research, we include subjective, self-report measures of infection risk in addition to our objective measure (i.e., state-level communicable disease mortality rates), which we predict will correlate significantly with each other.

In the current investigation, we report two studies that test the relationships between current objective pathogen risk, perceived infection risk, and pathogen and sexual disgust. A pilot study in El Salvador is also located in the Supplemental Materials, and in Tables S1 and S2; a power analysis determined the study was underpowered and, as a result, most results were non-significant.

In Study 1, we test this hypothesis in a large global sample, and measure how recent, state-level infection mortality rates influence disgust levels. Because the BIS is hypothesized to have evolved to respond to perceived pathogen cues, we anticipate mortality rates (which reflect objective pathogen risk) will correlate significantly with perceived infection exposure, and that both variables will positively predict disgust.

In Study 2, we use the novel SARS-COV-2 pandemic environment to assess this relationship during a period in which people experienced a large increase in pathogen risk globally. In a second online global sample, we test our core hypothesis that state mortality rates from infection and perceived infection exposure positively predict disgust levels. We then translate these variables to target the 2020 SARS-CoV-2 pandemic by substituting perceived general infection exposure and infection mortality rates with perceived risk of catching SARS-CoV-2 and using objective state-level case rates in our analysis. SARS-CoV-2 (formerly HCoV-19 and commonly known as COVID-19) is highly communicable, stable for a number of hours in aerosols and on surfaces^29^. Common symptoms are dry cough, trouble breathing, infection, pneumonia, and headaches^30^; these symptoms are prevalent in both adults and children^31^. While SARS-CoV-2 is the seventh coronavirus recorded, it is one of the most severe, among SARS-CoV and MERS-CoV, leading to an official global pandemic in March 2020, and has been confirmed in at least 213 countries and territories as of June 2020^32^. SARS-CoV-2 is highly infectious and poses a risk to individuals worldwide; thus, we use this unique event to address our hypotheses. We predict that perceived risk of contracting SARS-CoV-2 and state rates of SARS-CoV-2 cases will also positively predict both domains of disgust.

## Study 1

### Methods

#### Participants

This study was approved by the Boston University Institutional Review Board (IRB); all protocols were followed in accordance with the IRB and participants gave informed consent. We recruited 548 participants from Amazon’s Mechanical Turk (MTurk) to complete the above measures as part of a larger study on human health and mating prior to the SARS-CoV-2 pandemic. After accounting for failed attention checks, repeat IP addresses, and missing listwise data, 361 participants remained. Participants reported the country and state where they currently resided. The participants ranged in age from 18–63 years old (*M*_age_ = 26.12. *SD* = 5.50). Participants were from 23 countries; the two largest groups were India (*n* = 125; 50 women) and the US (*n* = 220; 116 women). This sample size provides sufficient power for a medium effect size (d = 0.03), according to Cohen’s *d* conventions^33^, based on an analysis in G*Power^34^ (d = 0.3, *α* = 0.05, power = 0.80, N = 101). Participants were compensated 1.50 USD after completing the study.

### Measures

#### Perceived infection exposure

Participants were asked to think of an average day in the past year and report the proportion of people in their community, family, and place of work who exhibited signs of infection on a scale of 0 to 100 (0 = no one exhibiting infection, 100 = everyone exhibiting infection). Studies show that humans detect disease cues in others reliably^4,25^ and remember diseased individuals, possibly to avoid future contact^35^. The description of an infectious disease was provided as “a disease caused by the entrance into the body of organisms (such as bacteria, protozoans, fungi, or viruses) which grow and multiply there.” Participants reported the proportion of people in their community, family, and place of work separately, yielding three different scores. These were then averaged to compute total perceived infection exposure which was used in the analyses. See Table 1 for descriptive statistics for each study sample; Cronbach’s α reflects the α across the three distinct items prior to summing the final score.

**Table 1:** Descriptive statistics of perceived infection exposure variable.

	Range	Mean	St. Dev	Cronbach’s α
Study 1	0.00–100.00	27.53	19.59	.83
Study 2	0.00–100.00	30.79	27.59	.94

#### Three Domains of Disgust Scale

Tybur and colleagues^23^ developed the TDDS as a scale to measure the aforementioned three domains of disgust. The measure contains 21 items, which participants are asked to rate on a scale of not at all disgusting (0) to extremely disgusting (6). The scale has been administered to several diverse populations, has demonstrated good internal consistency (α = 0.83 – 0.89), and has a coherent factor structure^15^. Example items for each domain are as follows: “stepping on dog poop” (pathogen disgust), “watching a pornographic video” (sexual disgust), and “stealing from a neighbor” (moral disgust). The seven items under each domain were averaged to give each participant three scores. We included moral disgust in our analyses, however due to its lack of relevance to the research questions, we focus on pathogen and sexual disgust in the Results sections. Results from regressions including moral disgust can be found in Tables S3, S5, S6.

#### State-level infection mortality

We calculated state-level death rates using Global Burden of Disease data^36^. Death rates per 100,000 people were included for all of respondents’ country of residence and included all infectious diseases listed: HIV/AIDS, tuberculosis, diarrheal, intestinal, STIs, and lower respiratory. Death rates from each disease were summed for each country in 2017 (the most recent data available).

### Data analysis

Study 2 data had the potential to violate the independence of observations assumption of linear modeling because participants were nested in states and countries. We examined intra-class correlations (ICCs) for our disgust variables and found a small amount of the variance was between countries (ICCs ranged from 0.008 to 0.110). At the state level, there was an average of five participants per state and substantial variance in disgust was between states (ICCs ranged from 0.160 to 0.311). Because even a small degree of clustering can impact standard errors if unaccounted for^37^, we controlled country using a fixed effect and used the complex samples package in SPSS to adjust standard errors for clustering at the state level. As in Study 1, we also controlled sex and age. Again, all data appeared to be normally distributed according to measures of kurtosis and skewness. Cronbach’s alpha level for all three domains of disgust resembled previous studies (*α*: moral = 0.87, sexual = 0.85, pathogen = 0.82; Tybur et al., 2009). We used *t*-tests to analyze significant mean differences between men and women, as well as between the Indian and US subsamples.

## Results

**Figure 1:**
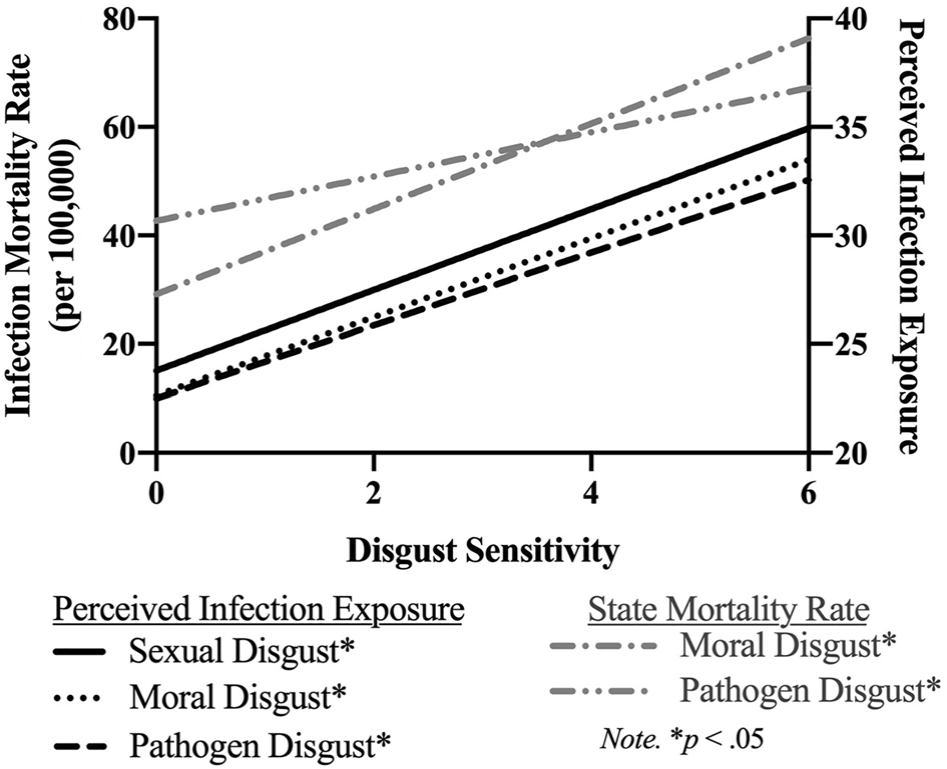
Study 1: relationship between perceived infection exposure, objective infection mortality rates, and disgust sensitivity.

Perceived infection exposure and state infection mortality correlated significantly (*r* = 0.40, *p* < 0.001). Variance Inflation Factors (VIF) indicated multicollinearity was not high enough to inflate standard errors (VIF = 1.14); therefore, we simultaneously entered both perceived and objective mortality risk in the models. Perceived infection exposure significantly predicted both domains of disgust (pathogen: *b* = 0.02, *SE* < 0.01, β = 0.21, *p* < 0.01; sexual: *b* = 0.01, *SE* < 0.01, β = 0.32, *p* < 0.01). State infection mortality rates significantly predicted pathogen (*b* = 0.01, *SE* < 0.01, β = 0.35, *p* = 0.03), but not sexual disgust. See Fig. 1 and Table S3 for all results.

**Figure 2:**
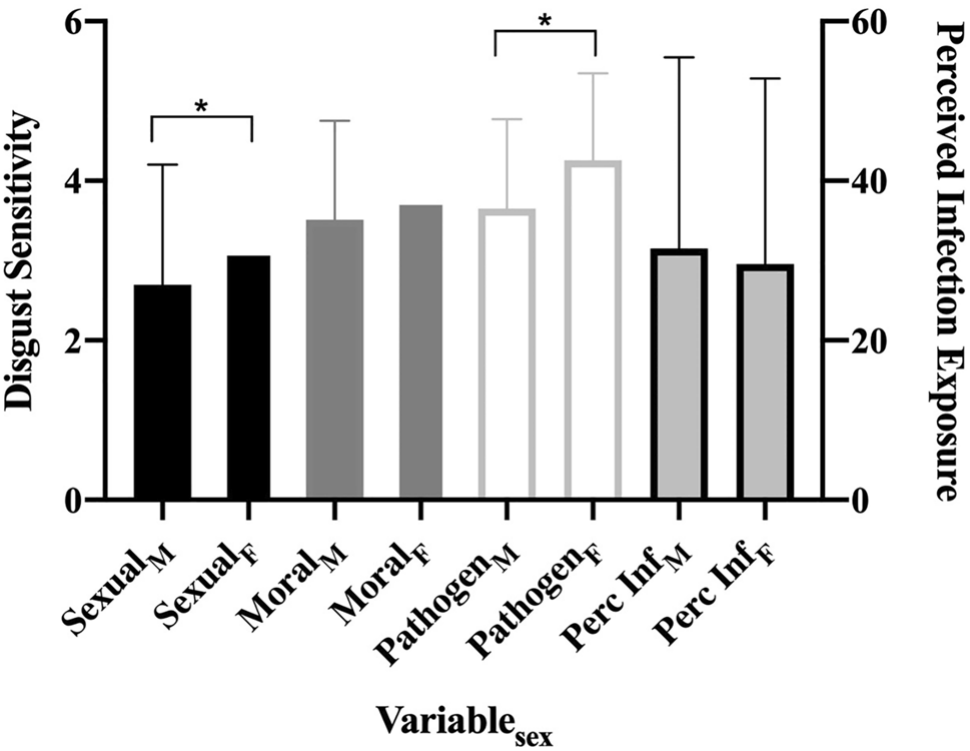
Study 1: sex differences. *Note.* Bars are standard errors. Perc Inf = perceived infection exposure. *p < .05.

In line with Al-Shawaf and colleagues^11^, there were significant sex differences, such that women reported greater sexual [t(357) = 2.91, *d* = 0.31, *p* < 0.01] and pathogen [t(357) = 5.15, *d* = 0.54, *p* < 0.01] disgust. There were no significant sex differences in moral disgust or perceived infection exposure. See Fig. 2 and Table S4 for all results. India was significantly higher than the US in sexual disgust [t(218) = 4.01, *d* = 0.55, *p* < 0.01] and perceived infection exposure [t(218) = 5.60, *d* = 0.75, *p* < 0.01]. The two subsamples did not differ on pathogen [t(218) = 0.97, *d* = 0.13, *p* = 0.33] or moral disgust [t(218) = 1.28, *d* = 0.18, *p* = 0.20].

## Discussion

In Study 2, perceived infection exposure and mortality rates were positively correlated, lending support to the conceptual validity of our perceived infection exposure variable. Perceived infection exposure positively predicted both domains of disgust. In addition, state mortality rates from infection contributed unique variance in predicting pathogen, but not sexual disgust.

Building on these results, Study 2 aimed to test the hypothesis that perceived infection exposure correlates with greater disgust sensitivities in the context of the SARS-CoV-2 pandemic. We replicated the methods from Study 1 in a larger sample and translated perceived infection exposure and infection mortality rates to perceived risk of contracting SARS-CoV-2 and state rates of SARS-CoV-2 cases, respectively. We assessed the association between SARS-CoV-2 risk (subjective and objective) and individual differences in pathogen and sexual disgust sensitivities.

## Study 2

### Methods

#### Participants

This study was approved by the Boston University Institutional Review Board (IRB); all protocols were followed in accordance with the IRB and participants gave informed consent. We recruited 1495 online participants using MTurk during the first week of April 2020. After accounting for failed attention checks, repeat IP addresses, and missing listwise data, we were left with 821 participants (257 women). This sample size also provides sufficient power for a medium effect size (d = 0.03), according to Cohen’s *d* conventions^33^, based on an analysis in G*Power^34^ (d = 0.3, *α* = 0.05, power = 0.80, N = 101). All participants reported being familiar with SARS-CoV-2, and completed the measures used in Study 1 as part of a larger study measuring the influence of the SARS-CoV-2 pandemic on behavioral and psychological shifts. The participants ranged in age from 18–72 years old (*M*_age_ = 31.55, *SD* = 9.91); 210 (67 women) were from Brazil, 205 (44 women) were from India, 178 (57 women) were from Italy, and 228 (90 women) were from the US. These countries were chosen to be diverse in culture, economic development, and communicable disease prevalence, but also because they varied in their SARS-CoV-2 progression. For example, when these data were collected, Italy was past its first peak, the US was still nearing its first peak, and Brazil and India were still leading up to their first peaks^38^.

#### Measures

Three Domains of Disgust Scale, Perceived Infection Exposure, and State-Level Infection Mortality were used.

#### Perceived infection exposure: SARS-CoV-2

Perceived risk of SARS-CoV-2 was measured using the question “What do you think the risk is that you will catch the Coronavirus (COVID-19)?” Participants responded using a scale from 1 (no risk) to 5 (very high risk).

#### Rates of infection: SARS-CoV-2

Rates of SARS-CoV-2 were calculated per state using SARS-CoV-2 cases numbers on April 26, 2020 from the European Centre for Disease Prevention and Control and the Johns Hopkins Coronavirus Resource Center. Rates were then calculated from state populations, yielding number of SARS-CoV-2 cases per 100,000.

### Data analysis

We followed the same steps as Study 1 to test the relationships between perceived infection exposure, mortality rates, and all domains of disgust. We controlled for age and sex. Examining the intra-class correlations (ICCs) for our disgust variables, we found a small amount of the variance was between countries (ICCs ranged from 0.002 to 0.113). At the state level, there was an average of 8.71 participants per state and substantial variance in disgust was between states (ICCs ranged from 0.133 to 0.208). Notably, ICCs were very similar between this study and Study 1 and their order was the same, with pathogen disgust varying least between states and countries and sexual disgust varying most at both levels. For the same reason as in Study 1, we controlled country by including fixed effects for country-level mean disgust levels and used the complex samples package in SPSS to adjust standard errors for clustering at the state level. Data were initially analyzed at the state or province level for all countries. Then, we substituted local infection mortality and perceived infection exposure variables for state-level SARS-CoV-2 case rates and perceived risk of contracting SARS-CoV-2, respectively, for a general linear model to predict each domain of disgust, analyzing the data from US, India, Brazil, and Italy.

A one-way ANOVA was used to assess country differences in all domains of disgust, perceived infection exposure, and SARS-CoV-2 infection rates. Cronbach’s alpha level for all three domains of disgust resembled previous studies (*α*: moral = 0.87, sexual = 0.85, pathogen = 0.83^23^).

## Results

**Figure 3:**
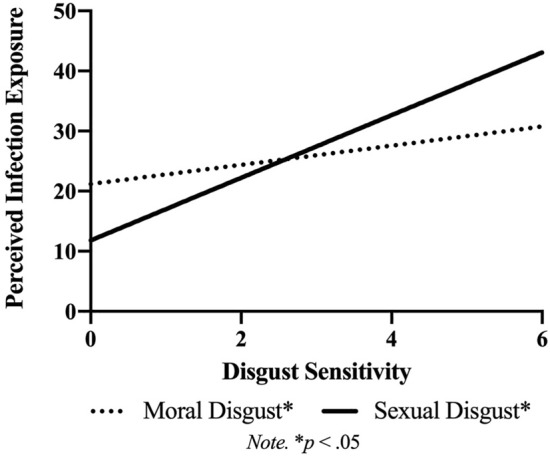
Study 2: relationship between perceived infection exposure and disgust sensitivity.

First, we sought to replicate findings from Study 1. Perceived infection exposure and state infection mortality rates again correlated significantly (*r* = 0.33, *p* < 0.01). VIFs indicated multicollinearity was not high enough to inflate standard errors (VIF = 1.15), so perceived and objective infection risk were simultaneously entered into models with sex and age. When all countries were included in the sample, perceived infection risk significantly predicted sexual disgust (*b* = 0.02, *SE* < 0.01, β = 0.38, *p* < 0.01), but not pathogen (*b* =  < 0.01, *SE* < 0.01, β = 0.23, *p* = 0.34) disgust, partially replicating Study 1. State infection mortality rates did not explain any additional variance in disgust, unlike results found for Study 1. See Fig. 3 and Table S5 for all results.

Using the SARS-CoV-2 pandemic as a specific event to test our hypotheses, we then substituted perceived risk of contracting SARS-CoV-2 and state rates of SARS-CoV-2 in a second model. Perceived risk of contracting SARS-CoV-2 and state rates of SARS-CoV-2 correlated significantly (*r* = 0.14, *p* < 0.01; VIF = 1.02).

**Figure 4:**
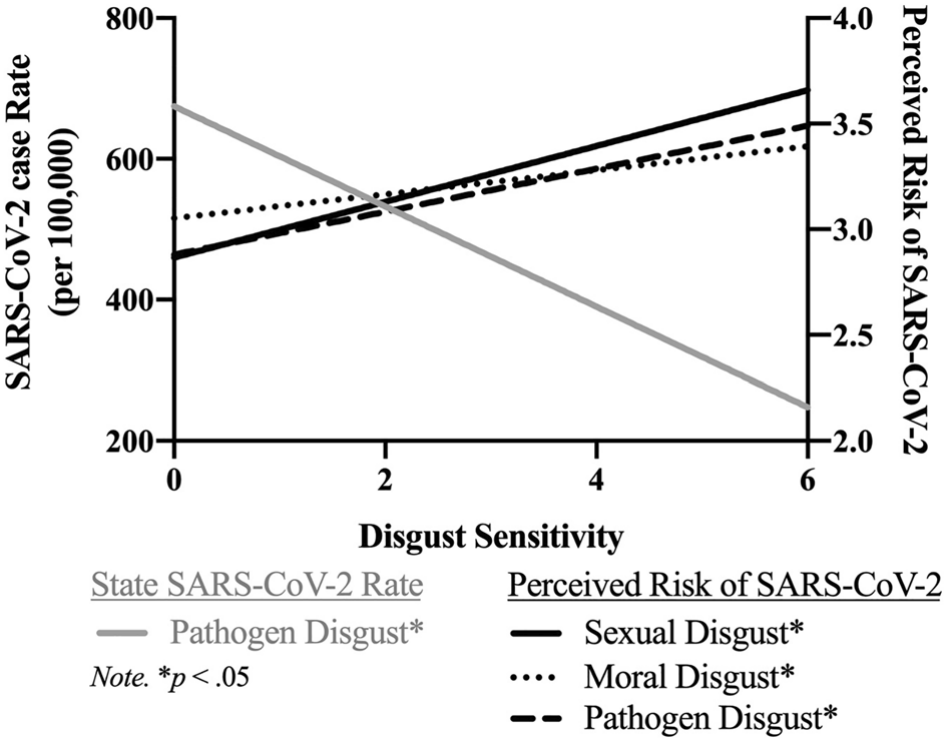
Study 2: relationship between perceived SARS-CoV-2 risk, objective SARS-CoV-2 rates, and disgust sensitivity.

Perceived risk of contracting SARS-CoV-2 was a significant positive predictor of both domains of disgust, in line with our hypotheses and Study 2’s results (sexual: *b* = 0.18, *SE* = 0.04, β = 0.14, *p* < 0.01; pathogen: *b* = 0.15, *SE* = 0.04, β = 0.14, *p* < 0.01). COVID state case rates significantly predicted pathogen disgust, however, in the *opposite* direction of our predictions (*b* = -0.22, *SE* = 0.04, β = -0.10, *p* < 0.01). See Fig. 4 and Table S6 for all results.

**Figure 5:**
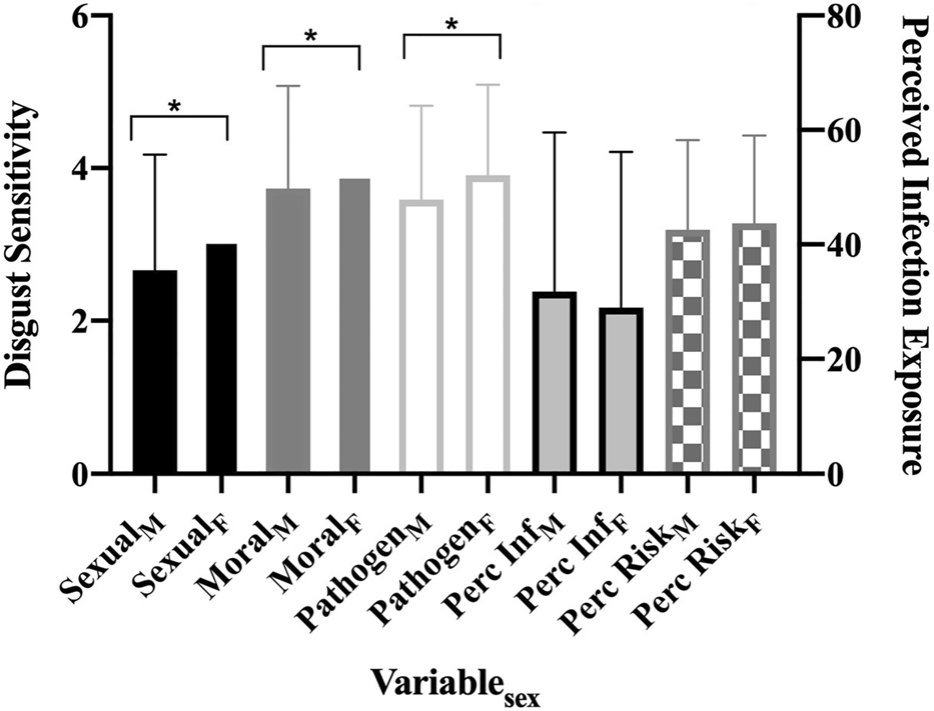
Study 2: sex differences. *Note*. Bars are standard errors. Perc Inf = perceived infection exposure; Perc Risk = perceived risk of catching SARS-CoV-2. *p < .05.

**Figure 6:**
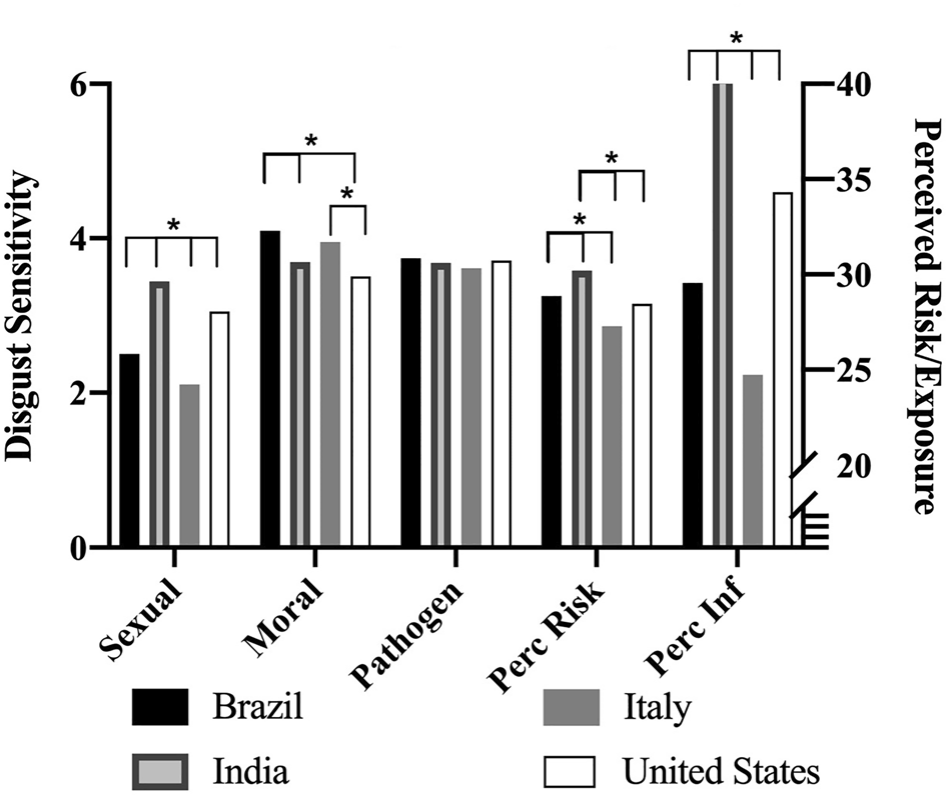
Country means of variables. *Note*. Perc Inf = perceived infection exposure; Perc Risk = perceived risk of catching SARS-CoV-2. *p < .05.

Women exhibited greater sexual disgust [t(821) = 3.99, *d* = 0.31, *p* < 0.01], pathogen disgust [t(820) = 3.5, *d* = 0.27, *p* < 0.01], and moral disgust [t(821) = 2.13, *d* = 0.17, *p* = 0.03]. There were no significant sex differences in perceived infection exposure or perceived risk of SARS-CoV-2 (Fig. 5; Table S7). When comparing respondents between countries, there were significant differences in sexual disgust [F(3) = 34.79, *p* < 0.01], moral disgust [F(3) = 8.18, *p* < 0.01], perceived infection exposure [F(3) = 59.57, *p* < 0.01], and perceived risk of SARS-CoV-2 [F(3) = 13.02, *p* < 0.01], but not pathogen disgust [F(3) = 0.40, *p* = 0.75]. Tukey’s post-hoc tests revealed that all groups’ mean significantly differed from each other in sexual disgust (Brazil_*M*_ = 2.5, *SD* = 1.38; India_*M*_ = 3.44, *SD* = 1.43; Italy_*M*_ = 2.11, *SD* = 1.25; US_*M*_ = 3.05, *SD* = 1.46; all *p*’s = 0.03 – < 0.01). For moral disgust, Brazil (*M* = 4.1, *SD* = 1.36) differed significantly from India (*M* = 3.69, *SD* = 1.33) and the US (*M* = 3.51, *SD* = 1.5), and Italy (*M* = 3.95, *SD* = 1.16) differed from the US (all *p*’s = 0.01 – < 0.01). All groups’ mean significantly differed from each other in perceived infection exposure (Brazil_*M*_ = 24.43, *SD* = 21.95; India_*M*_ = 48.32, *SD* = 27.01; Italy_*M*_ = 15.95, *SD* = 17.58; US_*M*_ = 32.85, *SD* = 30.33; all *p*’s = 0.01 – < 0.01). For perceived risk of contracting SARS-CoV-2, Brazil (*M* = 3.25, *SD* = 1.15) differed significantly from India (*M* = 3.58, *SD* = 1.09) and Italy (*M* = 2.86, *SD* = 1.17), and India and Italy also differed from the US (*M* = 3.15, *SD* = 1.16; all *p* = 0.05 – < 0.01). See Fig. 6. Finally, there were significant differences in state case rates between countries [F(3) = 279.49, *p* < 0.01]. Tukey’s post-hoc tests revealed that all group means significantly differed from each other (Brazil_*M*_ = 29.98, *SD* = 78.63; India_*M*_ = 1063.99, *SD* = 581.54; Italy_*M*_ = 374.24, *SD* = 251.73; US_*M*_ = 333.84, *SD* = 390.79; all *p*’s =  < 0.01), with the exception of Italy and the US.

## Discussion

In Study 1, we found that both perceived infection exposure and objective state-level mortality positively predicted pathogen disgust, whereas perceived infection exposure predicted sexual disgust. In Study 2, we examined these results in a larger sample, as well as extended our hypotheses to the SARS-CoV-2 pandemic. Perceived infection exposure positively predicted sexual disgust, replicating results from Study 2. Interestingly, pathogen disgust was not predicted by perceived infection exposure, similar to results found in the Pilot Study (located in the supplemental materials; Tables S1 and S2). In contrast to Study 1, state-level infection mortality rates did not predict any domain of disgust when included in the same model as perceived infection exposure, however the relationships were in the predicted positive direction. Overall, our results from Study 1 and 2 largely support the existence of a BIS, such that perceived infection exposure and objective mortality are correlated, and both predict disgust levels, possibly to promote pathogen-avoidant behaviors*.*

## General discussion

**Figure 7:**
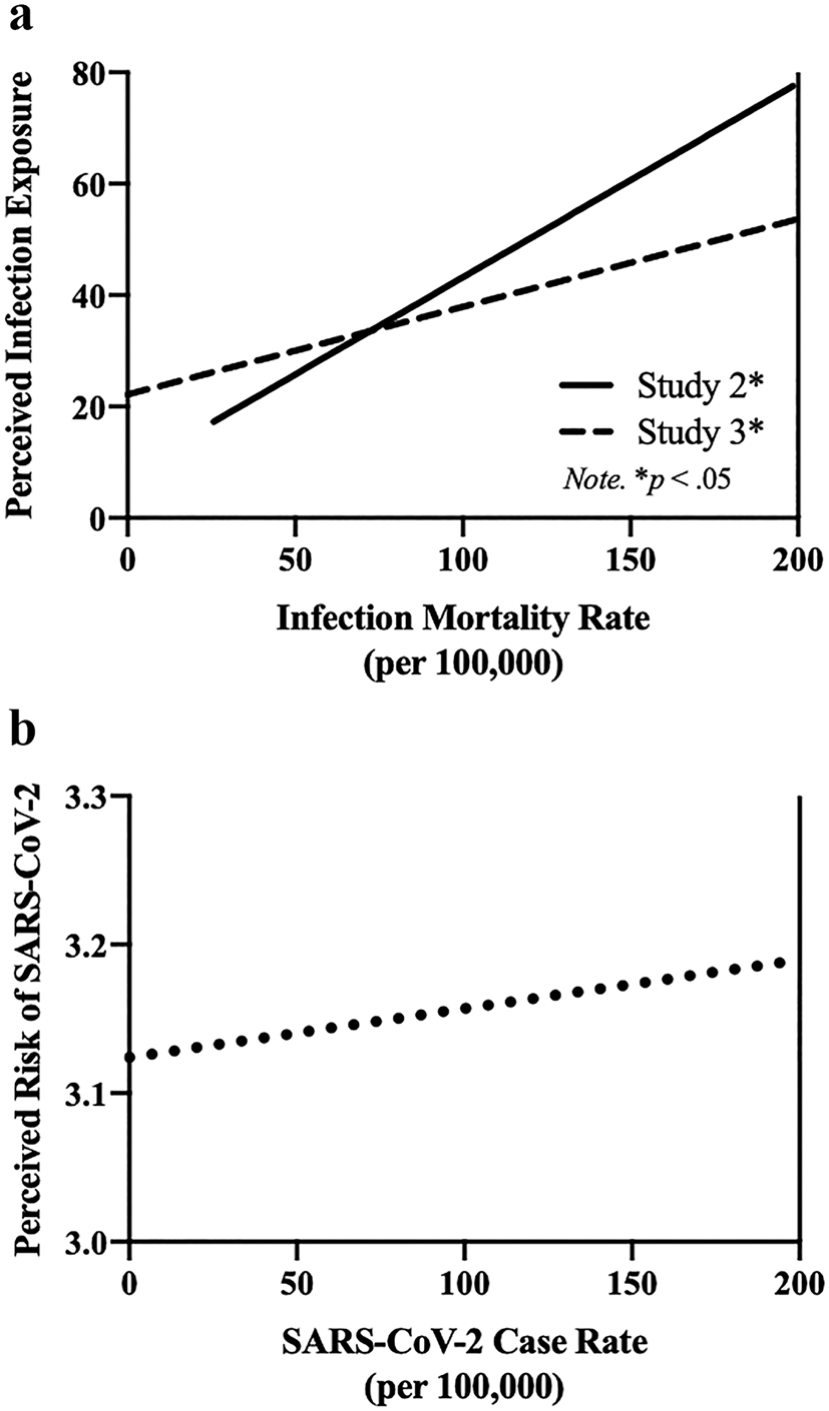
(**a**) Correlation of state infection mortality rate and perceived infection exposure. (**b**) Correlation of SARS-CoV-2 case rate and perceived SARS-CoV-2 risk*.

The goal of the present research was to address previously missing, foundational support for the behavioral immune system (BIS). To our knowledge, neither the relationship between current pathogen exposure and disgust, nor the correlation between objective and perceived pathogen risk have been addressed using ecologically valid measures in a global sample. This relationship is at the core of the hypothesized adaptive nature of disgust; that is, its role in activating the BIS and reducing exposure to communicable disease. Here, we show that perceived infection risk and local communicable disease mortality rates correlate with one another (Fig. 7a,b) and contribute unique variance in predicting domains of disgust. Together, these studies provide novel, theory-based findings to support the literature on the BIS and the adaptive function of disgust.

Previous studies attempting to address these relationships have only used the Index of Disease Prevalence^24^ (IDP) to measure environmental pathogen load. The IDP has two important limitations: it uses historical data and includes only nine infectious diseases. Using this index, Skolnick and Dzokoto^22^ found that in Ghana (a higher IDP country), participants had higher pathogen and moral disgust levels than in the US (a lower IDP country); sexual disgust was not examined in this study. Tybur and colleagues^23^ extended this analysis to include 30 countries, finding that the IDP predicted traditionalism; however, it was not significantly related to pathogen disgust and associations with sexual and moral disgust were not tested. One potential explanation for these mixed results may be that the IDP is not a measure of *current* pathogen load. Here, we use the most recent available state-level infectious disease mortality rates as a measure of prevailing, local infection risk. Our findings using SARS-CoV-2 infection risk underscore the idea that the BIS is likely responsive to contemporary infection risk, rather than historical trends.

In both studies, perceived infection exposure and communicable disease mortality rates were moderately positively correlated (see Fig. 7a,b and Table 2). These results indicate that individuals’ perceptions are reliable estimates of their actual risk of mortality due to infectious disease, in line with our prediction and the BIS framework. In order for the BIS to be effective, perception of disease cues should be derived from cues of actual pathogen risk in the local environment. Perception has been shown to be a trusted mechanism of detecting others’ infection risk through cues such as olfaction^25,26^, appearance^26,27^, and body motion^28^; nonetheless, these previous studies have been experimental, and this relationship has yet to be explored as a function of naturally occurring environment variation or in relation to disgust levels. In the current investigation, we addressed this gap in the literature, reporting the critical finding that perception is indeed correlated with local pathogen risk (i.e., communicable disease mortality risk), and thus a reliable indicator of objective infection threat.

**Table 2:** Summary of infection variable correlations.

**Study 1**	State Infection Mortality Rates
Perceived Infection	.40**
**Study 2**	
Perceived Infection	.33***
	State SARS-CoV-2 Rates
Perceived SARS-CoV-2 risk	.14**

**p < .01; ***p < .001.

Further, in both studies, both perceived infection risk and objective communicable mortality risk predicted domains of disgust, controlling for sex, age, and country of residence. In Study 1, perceived infection risk and mortality rates each explained unique variance in pathogen disgust, whereas only perceived risk predicted sexual disgust. In other words, perceived infection exposure is related to state-level mortality rates from infection, and both may independently influence pathogen disgust. Although perceived risk likely varies in response to local, objective disease risk, these findings suggest that objective mortality risk may also influence disgust via pathways other than perceived risk (e.g., cultural differences such as traditionalism^23^).

In Study 2, only perceived infection risk predicted sexual disgust sensitivity; that is, objective mortality rates did not predict any variance in disgust when perceived risk was controlled (Table 3). In contrast to our predictions and Study 1’s results, neither objective mortality nor perceived infection risk predicted pathogen disgust in Study 2. Because these data were collected during the SARS-CoV-2 pandemic, risk of contracting SARS-CoV-2 may have dominated perceptions compared to non-SARS-CoV-2 mortality (as measured in Study 1). Therefore, we then substituted perceived risk of SARS-CoV-2 infection as well as state case rates in SARS-CoV-2 in the models predicting each domain of disgust. Perceived risk of SARS-CoV-2 positively predicted all domains of disgust, in line with our hypotheses and results from Study 1. This accords with the idea that one’s perception of their environment may be more influential than “objective” measures; that is, the BIS should be more influenced by one’s perception of their own vulnerability to diseases^1^. Our results are also broadly consistent with the possibility that disgust indirectly reflects objective measures; that is, perceptions might mediate the effect of the latter on the former.

**Table 3:** Summary of results: standardized beta values from models presented in Tables S3, and S5.

	Pathogen disgust	Sexual disgust
**Study 1 (N = 232)**		
Perceived Infection	.21**	.32***
Mortality Rates	.35*	.03
**Study 2 (N = 821)**		
*Model* *1*		
Perceived Infection	.23	.38***
Mortality Rates	.30	.25
*Model* *2*		
Perceived SARS-CoV-2 risk	.14***	.14**
SARS-CoV-2 rates	-.10***	-.06

*p < .05; **p < .01; ***p < .001.

Interestingly, in Study 2, SARS-CoV-2 case rate *negatively* predicted pathogen disgust; that is, as case rate increased, pathogen disgust decreased. Although this was against our predictions, the unique context of the pandemic may yield unpredicted outcomes^39^. Since the pandemic affected humans globally, it is possible that disgust sensitivity levels rose across populations. However, this study focuses on state-level differences, and may capture nuances not seen in the global pattern. Also at the state-level, disgust sensitivity, and thus avoidance behaviors, may serve to protect some individuals better than others. For example, if resources are limited, increasing disgust may be too costly. Future studies can better assess this question using longitudinal data.

Since SARS-CoV-2 is a respiratory disease, and visual cues of the illness are not always present, it is possible that pathogen cues to which the BIS is attuned are not always exhibited by infected individuals^39^; this unexpected result may be partially explained by objective case rates not triggering the BIS. Alternatively, perceived risk may be based on those exhibiting visual and auditory cues, which would trigger the BIS. Additionally, increased case rates may lead to stricter lockdown and protocols (e.g., mask-wearing), causing individuals to feel safer and less disgusted while in their home bubble. Indeed, when mask-wearing may create a (potentially false) sense of safety^40^.

In both studies, participants’ country of residence mean disgust level was a significant predictor of sexual, but not pathogen disgust (see Table S3, S5, and S6). That is, different countries differed in their mean sexual disgust responses, but not pathogen disgust, and this predicted unique variance in individual’s reported disgust. In designing Study 2, we sought to collect data from several culturally and economically distinct countries. Variation across samples and countries underscores the need to survey diverse populations, and to address population-level variation in analyses and in sample descriptions^41,42^. The present studies add to the small, but growing literature on disgust utilizing cross-cultural samples. The majority of previous disgust studies only included university undergraduates from a single university^11,14,15,43–52^, or online samples^12,18,53–55^, with some online samples limited to the US^13,15,17^. Importantly, none of these studies report the ethnicity, nationality, or geographic breakdown of the participants. Here, we compare our hypotheses across diverse samples to assess the generalizability of the BIS framework and to explore the functional flexibility in disgust. Indeed, we see that all four countries in Study 2 differ in sexual disgust, perceived risk of SARS-CoV-2, and perceived infection exposure (Fig. 6). Nonetheless, the relationship between perceived infection exposure and objective infection mortality influence disgust consistently across the samples. Thus, our results suggest that infection risk and exposure contribute to variation in disgust levels across different environments, supporting the claim that disgust evolved to aid humans in detecting and avoiding pathogens^4,9,56^. We propose future work include more diverse samples and report demographics more transparently in order to assess the generalizability of results and their implications.

Finally, our study consistently found that women experience more disgust than men. These results contribute to a growing body of research suggesting that women engage in protective strategies due to their increased immunological vulnerability to pathogenic threats throughout their reproductive lifespans^57–61^. Indeed, research shows that women are consistently higher in disgust across all domains^11,15,55^, which our results largely support. Interestingly, there were no significant sex differences in any sample in perceived infection exposure or perceived SARS-CoV-2 risk. Because our results indicate that disgust sensitivity and perceived infection risk are closely related, the lack of sex difference in perceived infection risk suggests that sex differences are specific to disgust and not a consequence of upstream predictors like perceived infection risk. This suggests domain specificity in disgust psychology^4^.

### Future directions

Study 1 utilized the most recent infection mortality rates available, which were assessed in 2017^36^; future research could build on these findings by reassessing these relationships as new infection mortality rates are released. Second, because our data make it difficult to definitively point to a direction of causation^62^, future work using a variety of methods, including twin studies^63^, genetic data^64^ and longitudinal data collection, would continue to advance our understanding of the relationship between infection risk and disgust. Reverse causation is possible; disgust may heighten individuals’ attentiveness to pathogen cues, or the relationship between perception of pathogen risk and disgust may be bidirectional.

Future studies should also aim to disentangle the influence of local pathogen risk from other factors that influence disgust. For example, both sexual arousal and mating strategy have been found to be significant predictors of disgust^11,12,44,47,65^. Additionally, political and religious conservatism have been related to pathogen avoidance and disgust, although with varying support^17,23,52,66–68^. Researchers have also suggested that the intensity with which one reacts to pathogen cues should be dependent on their vulnerability^1^. We measure vulnerability here through environmental risk prevalence; however another source of risk is individuals’ health status or infection vulnerability, which has been associated with disgust^69–71^.

## Conclusion

Several previous studies have found that high pathogen prevalence, both historical and experimentally induced, are associated with increased protective behavior^20,21^, out-group opposition^13,14^, and overall distancing from others^18^. In line with a hypothesized BIS, these behaviors should be preceded by a psychological shift; yet no studies have shown that high pathogen risk is associated with disgust. We propose that disgust may be a leading emotional shift triggered by environmental pathogen change, which was supported by our results. This shift in disgust would then lead to shifts in behaviors and psychology previously linked to disgust sensitivity (e.g.,^9,12,53,55^). Practically speaking, research on the relationship between disgust and pathogen protective behavior, especially in the context of SARS-CoV-2 or other pathogen outbreaks, could shed light on variation in health protective behavior (e.g., handwashing, mask-wearing, social-distancing, etc.) across populations.

## Supplementary Information

Supplementary Information.
