# Supplementary material for: The evolution of disgust for pathogen detection and avoidance
Source: Sci Rep. 2021 Jun 29;11:13468. doi: 10.1038/s41598-021-91712-3 (PMC8241835; doi:10.1038/s41598-021-91712-3)
Supplement: Supplementary file 1 — Supplementary Information. [file 41598_2021_91712_MOESM1_ESM.docx]

**Supplemental Materials**

**Pilot Study**

**Participants**

This study was approved by the Franklin and Marshall College Institutional Review Board (IRB); all protocols were followed in accordance with the IRB and participants gave informed consent. We recruited 100 participants from El Salvador to complete the Three Domains of Disgust Scale (TDDS) and Perceived Infection Exposure questionnaire during a larger study on environment and behavior prior to the SARS-CoV-2 pandemic. After excluding individuals with more than 5% missing data, there were 98 participants (58 women) included in the analyses. The participants ranged in age from 18-48 years old (*M*_age_ = 23.68, *SD* = 7.40). Most self-identified as Latin American (88%) or White (8%); fewer (< 1% each) identified as Asian, South East Asian, Arab West Asian, or Indigenous. Participants were recruited in person at a university in the municipality of Santa Tecla, although participants did not have to be students at the university to participate. Participants were compensated 3.00 USD after completing the study.

**Data Analysis**

We imported all data from Qualtrics into SPSS (Version 25.0) for analyses. We conducted multiple regressions with α = 0.05 to test the relationships between perceived infection exposure to predict each of three disgust domains. The predictor and outcome variables appeared to be normally distributed based on our inspection of the kurtosis and skewness statistics. Following previous research, which has identified sex differences in disgust levels^1–3^, we controlled for sex and age. We report differences in disgust and perceived
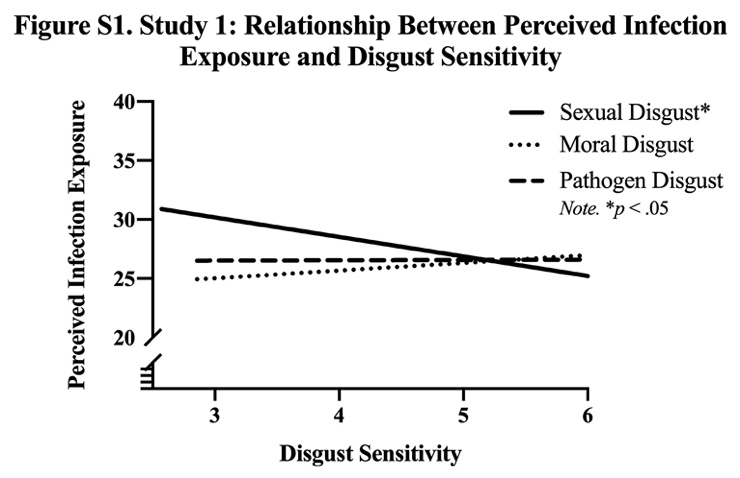
infection exposure in *t*-tests for each sample. Cronbach’s alpha level for sexual disgust resembled previous studies (*α* = .85; e.g.^1,2^), however pathogen and moral disgust were lower (*α*: moral = .71, pathogen = .56).

**Results**

Controlling for sex and age, perceived infection exposure was significantly *negatively* related to sexual disgust (*b* = -.02, *SE* = .01, β = -.21, *p* = .01), but was not associated with pathogen (*b* < .01, *SE* = .01, β = .03, *p* = .75) or moral disgust (*b* < -.01, *SE* = .01, β = -.03, *p* = .80). See Figure S1, as well as Table S1 for all statistics.


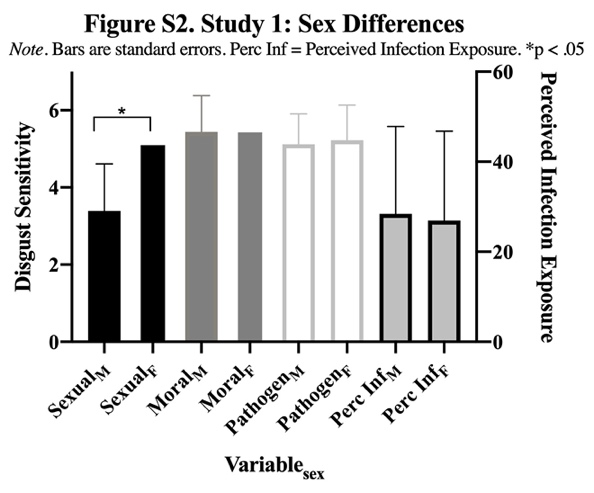
Whereas previous studies have reported a significant sex difference across all three domains of disgust, with women reporting higher levels^2^, our results show a significant difference only in sexual disgust, with women scoring higher [*t*(99) = 7.75, *d* = 1.62, *p* < .01]. There were no significant sex differences in perceived infection exposure. See Figure S2 and Table S2.

**Discussion**

These results do not support the hypothesis that higher perceived pathogen exposure is associated with higher disgust in all three domains. Sexual disgust was the only domain significantly predicted by perceived infection exposure, but the relationship was negative, opposite to our predictions. That is, we found that higher perceived infection exposure was associated with lower sexual disgust. Although the relationship between pathogen disgust and perceived infection exposure was in the predicted direction, it was not significant. Overall, this study yielded findings that suggested we needed to further investigate the research questions in a larger, more diverse sample. Further, adding in an objective measure of infection exposure may elucidate if our perceptions of our environment are accurate.

One possible explanation for this unpredicted finding is that Sample 1 was relatively small (*n* = 98) and culturally uniform. It is possible that perceived infection exposure may act as a mortality cue rather than pathogen warning in this sample due to El Salvador’s shorter national lifespan average^4^. One previous study found that disgust, specifically pathogen, lowered during a period of increased environmental harshness—potentially to take advantage of otherwise risky resources^5^. In addition, there was low reliability in the pathogen and moral domains of the TDDS, which can decrease power to detect an effect. In fact, a post-hoc power analysis using G*Power^6^ revealed that this study was slightly underpowered for a medium effect size (d = .03), according to Cohen’s *d* conventions^7^ (d = .3, *α* = .05, power = .80, N = 101). Therefore, this hypothesis should be repeated in a larger, more culturally diverse sample to assess if these results replicate across diverse populations, which is the aim of Study 1 and 2. Further, Study 1 and 2 also include a measure of objective state infection mortality rate, a reliable and salient indicator of actual infection risk.

**Pilot Study References**

1. Al-Shawaf, L., Lewis, D. M. G. & Buss, D. M. Disgust and mating strategy. *Evolution and Human Behavior* **36**, 199–205 (2015).

2. Tybur, J. M., Lieberman, D. & Griskevicius, V. Microbes, Mating, and Morality: Individual Differences in Three Functional Domains of Disgust. *Journal of Personality and Social Psychology* **97**, 103–122 (2009).

3. Al-Shawaf, L., Lewis, D. M. G. & Buss, D. M. Sex Differences in Disgust: Why Are Women More Easily Disgusted Than Men? *Emotion Review* 1–12 (2018) doi:10.1177/1754073917709940.

4. worldbank.org. Life expectancy at birth, total (years) | Data. https://data.worldbank.org/indicator/SP.DYN.LE00.IN.

5. Batres, C. & Perrett, D. I. Pathogen disgust sensitivity changes according to the perceived harshness of the environment. *Cognition and Emotion* 1–7 (2020) doi:10.1080/02699931.2019.1612735.

6. Faul, F., Erdfelder, E., Lang, A.-G. & Buchner, A. G*Power 3: A flexible statistical power analysis program for the social, behavioral, and biomedical sciences. *Behavior Research Methods* **39**, 175–191 (2007).

7. Cohen, J. *Statistical power analysis for the behavioral sciences*. (L. Erlbaum Associates, 1988).

**Supplemental Tables**

**Table S1.** Pilot Study: Multiple regressions analyzing the relationship between perceived infection exposure and each domain of disgust. *Note. r* = zero-order correlations between predictor and outcome variable*.* **p* < .05, ***p* < .01, ****p* < .001.

| Outcome | Predictors | *b* | SE | β | *p* | *r* |
| --- | --- | --- | --- | --- | --- | --- |
| **PATHOGEN DISGUST** | | | | | |  |
|  | Perceived Infection | <.01 | .01 | .03 | .75 | .043 |
|  | Sex | -.13 | .18 | -.08 | .47 | -.06 |
|  | Age | -<.01 | .01 | -.01 | .93 | -.01 |
|  | | | | | |  |
| **SEXUAL DISGUST** | | | | | |  |
|  | **Perceived Infection** | **-.02** | **.01** | **-.21** | **.01** | **-.21*** |
|  | **Sex** | **-1.75** | **.24** | **-.6** | **<.01** | **-.61***** |
|  | Age | .01 | .02 | .06 | .44 | -.05 |
|  | | | | | |  |
| **MORAL DISGUST** | | | | | |  |
|  | Perceived Infection | -<.01 | .01 | -.03 | .80 | .05 |
|  | Sex | -.12 | .10 | -.07 | .06 | -.05 |
|  | **Age** | **.01** | **.01** | **.09** | **.01** | **.30**** |

**Table S2.** Pilot Study: Means, SD, and effect sizes for sex differences in the domains of disgust and total perceived infection exposure. *Note.* **p* < .05, ***p* < .01, ****p* < .001.

| Scale | Women mean (SD) | | Men mean (SD) | Cohen’s *d* | |
| --- | --- | --- | --- | --- | --- |
| **TDDS** | | | | | |
| Sexual | 5.20 (1.11) | 3.39 (1.12) | | | 1.62*** |
| Pathogen | 5.22 (.91) | 5.12 (.79) | | | .12 |
| Moral | 5.54 (.92) | 5.44 (.94) | | | .11 |
|  |  |  | | |  |
| **PERCEIVED INFECTION** | | | | | |
|  | 26.91 (19.86) | 28.42 (19.41) | | | .08 |

**Table S3.** Study 1: Complex Samples General Linear Model analyzing the relationship between perceived infection exposure and state mortality rates and all domains of disgust. *Note. r* = zero-order correlations between predictor and outcome variable. **p* < .05, ***p* < .01, ****p* < .001.

| Parameter | | *b* | | SE |  | 95% Confidence Interval | | Hypothesis Test | | |  |
| --- | --- | --- | --- | --- | --- | --- | --- | --- | --- | --- | --- |
|  |  |  |  |  | β | Lower | Upper | *t* | df | *p* | *r* |
| **SEXUAL DISGUST** | |  | |  |  |  |  |  |  |  |  |
| (Intercept) | | | .80 | .76 |  | -.73 | 2.32 | 1.04 | 50 | .30 |  |
| **Perceived Infection** | | | **.02** | **<.01** | **.32** | **.01** | **.03** | **6.35** | **50** | **<.01** | **.45***** |
| State Mortality Rate | | | <.01 | <.01 | .03 | -.01 | .01 | .45 | 50 | .65 | .39*** |
| **Sex** | | | **.61** | **.15** | **.21** | **.31** | **.91** | **4.09** | **50** | **<.01** | **-.15**** |
| Age | | | .04 | .03 | .27 | -.03 | .10 | 1.02 | 50 | .32 | -.09 |
| **Country Disgust Mean** | | | **.77** | **.28** | **.26** | **.20** | **1.34** | **2.72** | **50** | **.01** |  |
|  |  | | |  |  |  |  |  |  |  |  |
| **MORAL DISGUST** |  | | |  |  |  |  |  |  |  |  |
| (Intercept) | | | 2.23 | .85 |  | .53 | 3.93 | 2.64 | 50 | .01 |  |
| **Perceived Infection** | | | **.01** | **<.01** | **.18** | **<.01** | **.02** | **2.89** | **50** | **.01** | **.20**** |
| **State Mortality Rate** | | | **.01** | **<.01** | **.30** | **.01** | **.02** | **4.27** | **50** | **<.01** | **.25***** |
| **Sex** | | | **.50** | **.14** | **.19** | **.23** | **.78** | **3.73** | **50** | **<.01** | **-.10** |
| Age | | | .01 | .04 | .07 | -.06 | .09 | .37 | 50 | .72 | .05 |
| **Country Disgust Mean** | | | **-.73** | **.25** | **-.27** | **-1.23** | **-.24** | **-2.97** | **50** | **.01** |  |
|  |  | | |  |  |  |  |  |  |  |  |
| **PATHOGEN DISGUST** | | | |  |  |  |  |  |  |  |  |
| (Intercept) | | | 3.12 | .63 |  | 1.85 | 4.38 | 4.94 | 50 | <.01 |  |
| **Perceived Infection** | | | **.01** | **<.01** | **.21** | **<.01** | **.01** | **2.69** | **50** | **.01** | **.17**** |
| **State Mortality Rate** | | | **.01** | **<.01** | **.35** | **<.01** | **.01** | **2.21** | **50** | **.03** | **.09** |
| **Sex** | | | **.64** | **.14** | **.28** | **.37** | **.92** | **4.68** | **50** | **<.01** | **-.26**** |
| Age | | | <.01 | .03 | .01 | -.05 | .05 | .03 | 50 | .98 | .03 |
| Country Disgust Mean | | | -.38 | .28 | -.16 | -.94 | .18 | -1.36 | 50 | .18 |  |

**Table S4.** Study 1: Means, SD, and effect sizes on the domains of disgust and total perceived infection exposure reported by sex. *Note.* **p* < .05, ***p* < .01, ****p* < .001.

| Scale | Women mean (SD) | | Men mean (SD) | Cohen’s *d* | |
| --- | --- | --- | --- | --- | --- |
| **TDDS** | | | | | |
| Sexual | 3.12 (1.34) | 2.68 (1.51) | | | .31** |
| Pathogen | 4.27 (1.11) | 3.67 (1.1) | | | .54*** |
| Moral | 3.79 (1.39) | 3.53 (1.27) | | | .20 |
|  |  |  | | |  |
| **PERCEIVED INFECTION** | | | | | |
|  | 29.54 (23.16) | 30.96 (23.85) | | | .06 |

**sssssssssss**

**Table S5.** Study 2: Complex Samples General Linear Model analyzing the relationship between perceived infection exposure and state mortality rates and all domains of disgust. *Note. r* = zero-order correlations between predictor and outcome variable. **p* < .05, ***p* < .01, ****p* < .001.

| Parameter | | *b* | | SE |  | 95% Confidence Interval | | Hypothesis Test | | |  |
| --- | --- | --- | --- | --- | --- | --- | --- | --- | --- | --- | --- |
|  |  |  |  |  | β | Lower | Upper | *t* | df | *p* | *r* |
| **SEXUAL DISGUST** | |  | |  |  |  |  |  |  |  |  |
| (Intercept) | | | .35 | .30 |  | -.25 | .94 | 1.16 | 96 | .25 |  |
| **Perceived Infection** | | | **.02** | **<.01** | **.38** | **.01** | **.02** | **9.66** | **96** | **<.01** | **.40***** |
| State Mortality Rate | | | <.01 | <.01 | .25 | -<.01 | <.01 | .09 | 96 | .93 | .22*** |
| **Sex** | | | **.52** | **.09** | **.16** | **.35** | **.69** | **6.09** | **96** | **<.01** | **-.14***** |
| Age | | | <.01 | <.01 | .07 | -.01 | .01 | .34 | 96 | .74 | .02 |
| **Country Disgust Mean** | | | **.61** | **.12** | **.21** | **.36** | **.85** | **.4.93** | **96** | **<.01** |  |
|  |  | | |  |  |  |  |  |  |  |  |
| **MORAL DISGUST** |  | | |  |  |  |  |  |  |  |  |
| (Intercept) | | | -2.71 | 1.01 |  | -4.71 | -.72 | -2.70 | 96 | .01 |  |
| **Perceived Infection** | | | **.01** | **<.01** | **.20** | **<.01** | **.01** | **2.25** | **96** | **.03** | **.03** |
| State Mortality Rate | | | <.01 | <.01 | .27 | -<.01 | <.01 | .12 | 96 | .91 | -.01 |
| Sex | | | .21 | .11 | .07 | -.01 | .42 | 1.92 | 96 | .06 | -.07* |
| **Age** | | | **.02** | **.01** | **.15** | **.01** | **.03** | **4.59** | **96** | **<.01** | **.09**** |
| **Country Disgust Mean** | | | **1.46** | **.24** | **.25** | **.97** | **1.94** | **6.01** | **96** | **<.01** |  |
|  |  | | |  |  |  |  |  |  |  |  |
| **PATHOGEN DISGUST** | | | |  |  |  |  |  |  |  |  |
| (Intercept) | | | .93 | 3.19 |  | -5.40 | 7.26 | .29 | 96 | .77 |  |
| Perceived Infection | | | <.01 | <.01 | .23 | -<.01 | .01 | .96 | 96 | .34 | .05 |
| State Mortality Rate | | | <.01 | <.01 | .30 | -<.01 | .01 | .29 | 96 | .77 | .01 |
| **Sex** | | | **.30** | **.08** | **.11** | **.15** | **.45** | **3.92** | **96** | **<.01** | **-.12***** |
| **Age** | | | **.01** | **<.01** | **.08** | **<.01** | **.02** | **2.95** | **96** | **<.01** | **.11**** |
| Country Disgust Mean | | | .59 | .87 | .02 | -1.15 | 2.32 | .67 | 96 | .50 |  |

**Table S6.** Study 2: Complex Samples General Linear Model analyzing the relationship between perceived SARS-CoV-2 risk and state SARS-CoV-2 rates and all domains of disgust. *Note. r* = zero-order correlations between predictor and outcome variable*.* **p* < .05, ***p* < .01, ****p* < .001.

| Parameter | | *b* | SE |  | 95% Confidence Interval | | Hypothesis Test | | |  |
| --- | --- | --- | --- | --- | --- | --- | --- | --- | --- | --- |
|  |  |  |  | β | Lower | Upper | *t* | df | *p* | *r* |
| **SEXUAL DISGUST** | |  |  |  |  |  |  |  |  |  |
| (Intercept) | | -.69 | .28 |  | -1.26 | -.12 | -2.41 | 94 | 0.2 |  |
| **Sex** | | **.50** | **.09** | **.16** | **.32** | **.69** | **5.40** | **94** | **<.01** | -.14*** |
| Age | | - <.01 | <.01 | .07 | -.01 | .01 | -.94 | 94 | 0.35 | .02 |
| **Country Disgust Mean** | | **1.05** | **.12** | **.36** | **.81** | **1.29** | **8.80** | **94** | **<.01** |  |
| COVID Case Rate | | -.17 | .10 | -.06 | -.35 | .02 | -1.75 | 94 | 0.08 | .12*** |
| **COVID Perceived Risk** | | **.18** | **.04** | **.14** | **.10** | **.26** | **4.52** | **94** | **<.01** | **.21***** |
|  |  | |  |  |  |  |  |  |  |  |
| **MORAL DISGUST** |  | |  |  |  |  |  |  |  |  |
| (Intercept) | | -1.86 | .93 |  | -3.71 | -.01 | -1.99 | 94 | 0.05 |  |
| Sex | | .17 | .10 | .06 | -.03 | .38 | 1.66 | 94 | 0.10 | -.07* |
| **Age** | | **.02** | **.01** | **.15** | **.01** | **.03** | **3.95** | **94** | **<.01** | **.09**** |
| **Country Disgust Mean** | | **1.21** | **.22** | **.26** | **.76** | **1.65** | **5.39** | **94** | **<.01** |  |
| COVID Case Rate | | -.09 | .06 | -.35 | -.21 | .02 | -1.57 | 94 | 0.12 | **-.11**** |
| **COVID Perceived Risk** | | **.13** | **.04** | **.11** | **.05** | **.22** | **3.14** | **94** | **<.01** | **.10**** |
|  |  | |  |  |  |  |  |  |  |  |
| **PATHOGEN DISGUST** | | |  |  |  |  |  |  |  |  |
| (Intercept) | | 3.25 | 3.53 |  | -3.76 | 10.25 | .92 | 94 | 0.36 |  |
| **Sex** | | **.27** | **.07** | **.10** | **.13** | **.41** | **3.74** | **94** | **<.01** | **-.12***** |
| **Age** | | **.01** | **<.01** | **.08** | **<.01** | **.02** | **2.79** | **94** | **.01** | **.11**** |
| Country Disgust Mean | | -.10 | .96 | -.06 | -2.02 | 1.81 | -.11 | 94 | 0.92 |  |
| **COVID Case Rate** | | **-.22** | **.08** | **-.10** | **-.37** | **-.07** | **-2.92** | **94** | **<.01** | **-.09**** |
| **COVID Perceived Risk** | | **.15** | **.04** | **.14** | **.07** | **.22** | **3.74** | **94** | **<.01** | **.13***** |

**Table S7.** Study 3: Means, SD, and effect sizes on the domains of disgust and total perceived infection exposure reported by sex. *Note.* **p* < .05, ***p* < .01, ****p* < .001.

| Scale | Women mean (SD) | | Men mean (SD) | Cohen’s *d* | |
| --- | --- | --- | --- | --- | --- |
| **TDDS** | | | | | |
| Sexual | 3.1 (1.32) | 2.66 (1.52) | | | .31*** |
| Pathogen | 3.91 (1.18) | 3.59 (1.23) | | | .27*** |
| Moral | 3.95 (1.41) | 3.73 (1.34) | | | .17* |
|  |  |  | | |  |
| **PERCEIVED INFECTION** | | | | | |
|  | 28.94 (27.21) | 31.77 (27.79) | | | .10 |
|  |  |  | | |  |
| **PERCEIVED SARS-CoV-2 RISK** | | | | |  |
|  | 3.28 (1.15) | 3.29 (1.18) | | | .08 |
